# Testing the relationship between intraspecific competition and individual specialization across both behavior and diet

**DOI:** 10.1002/ece3.7916

**Published:** 2021-07-17

**Authors:** Amaryllis K. Adey, Eric R. Larson

**Affiliations:** ^1^ Department of Biological Sciences University of Notre Dame Notre Dame Indiana USA; ^2^ Department of Natural Resources and Environmental Sciences University of Illinois Urbana Illinois USA

**Keywords:** crayfish, *Faxonius rusticus*, invasive species, stable isotopes

## Abstract

Individual specialization within populations is increasingly recognized as important in both ecology and evolution, but researchers working on intraspecific variation in behavior and diet infrequently interact. This may be because individual specialization on diet and behavior was historically difficult to investigate simultaneously on the same individuals. However, approaches like stable isotope analysis that allow hindcasting past field diets for laboratory organisms may provide opportunities to unite these areas of inquiry. Here, we tested the role of intraspecific competition on individual specialization through analysis of both behavior and diet simultaneously. We focused on intraspecific competition as a mechanism that might drive individual specialization of both diet and behavior. We conducted this study in Vilas County, Wisconsin, United States (US), using rusty crayfish *Faxonius rusticus* from six lakes across a relative abundance gradient. We conducted six assays to measure individual specialization of behavior and used stable isotope analysis to measure individual specialization of diet. We then related both measures of individual specialization to relative abundance of *F. rusticus* using linear and quadratic models. We found a unimodal relationship between intraspecific competition and individual specialization of diet for *F. rusticus,* likely because some preferred resources are unavailable to specialize on at the highest densities of this well‐studied crayfish invader. Conversely, we found greater support for a linear relationship between individual specialization of behavior and intraspecific competition, perhaps because specialization by behavior is not inherently resource‐limited. Our results show that dietary and behavioral specialization may exhibit different responses to increased intraspecific competition, and demonstrate a potential technique that can be used to investigate individual specialization of diet and behavior simultaneously for the same individuals and populations.

## Graphical abstract

Individual specialization within populations is increasingly recognized as important in both ecology and evolution, but researchers working on intraspecific variation in behavior and diet infrequently interact. Here, we tested the role of intraspecific competition on individual specialization through analysis of both behavior and diet simultaneously. We found a unimodal relationship between intraspecific competition and individual specialization of diet for populations of an invasive crayfish, but we found greater support for a linear relationship between individual specialization of behavior and intraspecific competition, perhaps because specialization by behavior is not inherently resource‐limited.

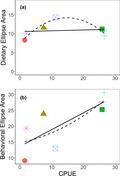

## 1 INTRODUCTION

Individuals within populations routinely differ by traits including morphology, behavior, and resource use like diet (Bolnick et al., 2011; Des Roches et al., 2018; Evangelista et al., 2019). The causes and consequences of this individual variation have been topics of high interest in both ecology and evolution (Araújo et al., 2011; Raffard et al., 2020; Yoder et al., 2010), emerging over recent decades as the study of the “ecology of individuals” or individual specialization (Bolnick et al., 2003). Historically, the study of individual specialization was strictly concerned with specialization on resources like food that could be directly linked by theory to population processes (Roughgarden, 1972, 1974). In this literature, individual specialization is characterized as high when broad population niches (i.e., the resource use of an entire population) are attributable to between‐individual differences in resource use rather than broad within‐individual resource use (Bolnick et al., 2003). Quantification of between‐ versus within‐individual resource use has progressed over time to apply methods like stable isotope analysis, which provides long‐term integration of individual diets in a single measure (Araújo et al., 2007; Bolnick et al., 2002). Concurrent with this growth of research on dietary individual specialization, the field of animal behavior similarly experienced heightened interest in individual‐level variation in behavior, often expressed as “behavioral syndromes” (i.e., when multiple behaviors are correlated within individuals; Sih et al., 2004) or “animal personalities” (i.e., when behaviors are consistent over time within individuals; Roche et al., 2016; Stamps & Groothuis, 2010). Yet, these dual emphases on behavioral and dietary variation within populations have infrequently interacted, symptomatic of a tendency for biology to become less integrated and more siloed over time (Kultz et al., 2013).

Toscano et al. (2016) recently proposed that intraspecific variation in behavior (i.e., animal personality) and diet (i.e., individual specialization) may often covary or be causally related. As just one example, animal personality traits like competitive hierarchies could be linked to outcomes in dietary resource acquisition (Briffa et al., 2015; but see Adey & Larson, 2020), with subsequent effects on population dynamics (Roughgarden, 1972, 1974). Yet, these possible links have remained underinvestigated, perhaps due to methodological challenges in studying behavior and diet simultaneously for the same individuals without affecting one or both measurable responses (Toscano et al., 2016). However, Glon et al. (2016a) proposed a solution to this methodological problem: Stable isotope analyses used to characterize dietary individual specialization might be applied to laboratory organisms assayed for behavior, particularly in cases where isotopic turnover rates are slow enough to reflect former in situ rather than current ex situ diets (Glon et al., 2016b; Vander Zanden et al., 2015). In these instances, individual behavioral variation inferred from laboratory assays can be compared back to individual specialization of diet from the field (Adey & Larson, 2020; Glon et al., 2016). Yet, to date, very few studies have attempted to link behavior and diet within or between populations of the same species using tools like stable isotope analysis (Toscano et al., 2016; Závorka et al., 2017).

Here, we sought to investigate behavioral and dietary individual specialization of organisms simultaneously in response to a gradient of intraspecific competition between populations. We use the term “individual specialization” in reference to both behavior and diet in order to simplify our writing, while acknowledging that the term individual specialization has historically referred specifically to limited resources like food (Bolnick et al., 2003; Roughgarden, 1972). Further, we have chosen to relate these measures of individual specialization to intraspecific competition in response to a priori expectations on the effects of intraspecific competition on both diet and behavior. First, with respect to individual specialization on diet, ecological theory predicts a linear relationship in which individual specialization should increase with increasing intraspecific competition (Futuyma & Moreno, 1988; Roughgarden, 1972). As population density increases, resources are depleted at accelerated rates; accordingly, individuals are predicted to become more specialized to reduce competitive pressure within the population (Bailey et al., 2013). However, some empirical studies have instead found a unimodal relationship where the greatest individual specialization occurs at moderate densities with decreasing specialization at both high and low densities (Abrams et al., 2008; Jones & Post, 2013; Mateus et al., 2016). This may occur because as resources are depleted at the highest population densities, many prey resources may become scarce and individual specialization may contract as organisms are forced to consume the similar, remaining prey. In a recent meta‐analysis, Jones and Post (2016) found that there were equal instances in which competition increased individual dietary specialization relative to those in which competition decreased individual dietary specialization.

Alternatively, individual specialization of behavior might increase continuously with intraspecific competition because behavior is not resource‐limited, unlike individual specialization of diet (Jones & Post, 2013; Svanbäck & Bolnick, 2007). While availability of some resources like food or habitat might decline at especially high intraspecific competition, individuals could continue to specialize behaviorally in how or when they exploit these resources (Toscano et al., 2016). However, behavioral specialization might also exhibit a unimodal relationship, with a peak in specialization at intermediate densities and a decline in specialization at highest densities, if particular behaviors are advantageous at high intraspecific competition. For example, some researchers have found emergence of behavioral syndromes at high population densities, in which certain correlated behaviors may be best for competing with conspecifics when resources are scarce (Pintor et al., 2009; Sih et al., 2004). Yet, relatively few studies have evaluated individual specialization of behavior over gradients of intraspecific competition, and none to our knowledge have compared dietary and behavioral specialization to each other over this same gradient simultaneously.

**FIGURE 1:**
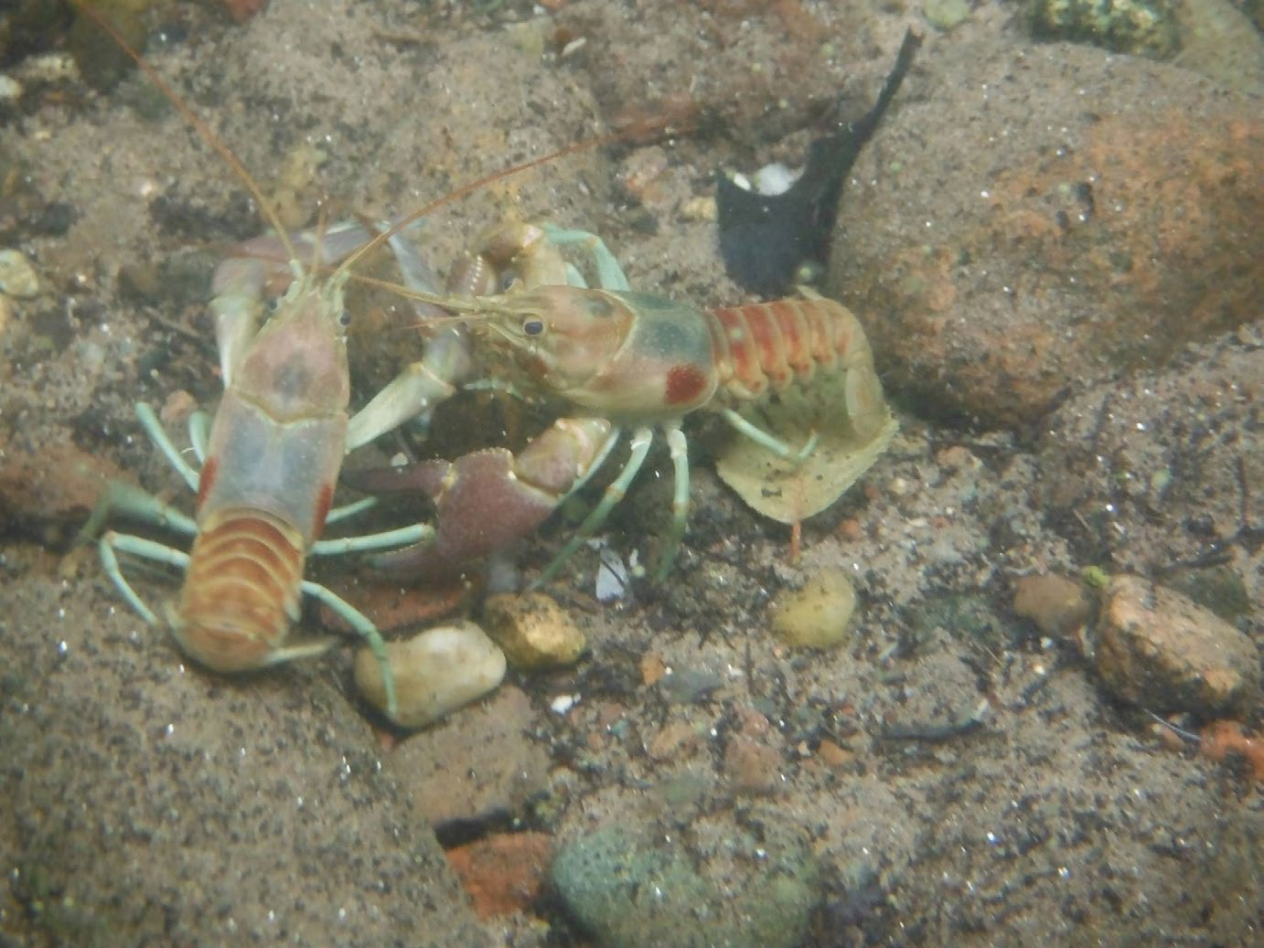
Rusty crayfish *Faxonius rusticus* in a lake of Vilas County, Wisconsin, US

We applied here the methodology of Glon et al. (2016a) to evaluate individual specialization of both diet and behavior concurrently for the rusty crayfish *Faxonius rusticus* over a gradient of relative abundance or intraspecific competition (Figure 1). We used invasive populations of *F*. *rusticus* in lakes where the species has been studied since the 1970s (Capelli & Magnuson, 1983), in part because this species has well understood behavioral ecology (e.g., Adey & Larson, 2020) and known isotopic turnover rates (Glon et al., 2016b). We hypothesized that *F*. *rusticus* dietary specialization should peak at intermediate relative abundances, as individual specialization for food might increase with increasing intraspecific competition until declining when some preferred prey resources are depleted by high densities of this organism (McCarthy et al., 2006). Alternatively, we hypothesized that *F*. *rusticus* behavioral specialization should increase linearly with increasing relative abundance, although behavioral specialization could decrease with increasing relative abundance if behavioral syndromes predominate where intraspecific competition is intense for this crayfish (Pintor et al., 2009). Our study is the first to empirically assess the effect of abundance on individual specialization using two different metrics of specialization on the same individuals and to demonstrate a methodology that could be more widely applied to reconcile disagreements between predicted and observed relationships between individual specialization and intraspecific competition.

## 2 METHODS

### 2.1 Study species and area

We conducted this study in Vilas County, Wisconsin, US, using *F*. *rusticus*, an invasive crayfish in the region. *Faxonius rusticus* is native to the Ohio River drainage of the Midwestern United States; however, it has been introduced throughout North America through multiple vectors including use as live bait and the biological supply trade (Larson & Olden, 2008; Lodge et al., 2000; Olden et al., 2006). *Faxonius rusticus* was first found in Wisconsin in the late 1960s and has since spread widely throughout this state (Capelli & Magnuson, 1983; Olden et al., 2006). *Faxonius rusticus* negatively impacts populations of its preferred prey resources, like snails and other macroinvertebrates, suggesting that this species likely experiences intraspecific competition at high relative abundances (McCarthy et al., 2006; Olsen et al., 1991; but see Messager & Olden, 2018 for discussion of density dependence in *F*. *rusticus*). Long‐term monitoring of populations of *F*. *rusticus* in northern Wisconsin provided us with an a priori understanding of *F*. *rusticus* relative abundances within these lakes (Larson et al., 2019). We aimed to collect crayfish from similar lakes (medium‐sized, mesotrophic) across a broad relative abundance gradient. We collected crayfish from Birch Lake, Boulder Lake, Papoose Lake, Presque Isle Lake, South Turtle Lake, and Spider Lake (Table 1).

**TABLE 1:** Attributes for each lake sampled for rusty crayfish *Faxonius rusticus* including catch‐per‐unit effort (CPUE) from our total baited trapping effort (24–30 traps per lake), lake size (ha), maximum depth (m), average 2018 Secchi disk depths (https://dnr.wi.gov/lakes/waterquality/Stations.aspx?location=64), and year invaded by *F. rusticus*

Lake	CPUE	Size (ha)	Max depth (m)	Secchi depth (m) ± *SD*	Year invaded
Birch Lake	1.5 (1, 2, 7)	205	14	1.8 ± 0.2	<1975
Boulder Lake	7.5 (6, 7, 25)	212	7	2.0 ± 1.2	<1975
Papoose Lake	25.9 (26, 32, 38)	173	20	4.2 ± 0.6	<1975
Presque Isle Lake	26.5 (29, 44, 56)	471	31	7.6 ± 1.2	<1975
South Turtle Lake	11.2 (8, 9, 13)	189	12	2.0 ± 0.4	<1975
Spider Lake	2.0 (4, 6, 7)	110	6	3.5 ± 0	<1987

Note

We report three CPUEs of individual trap locations where we collected *F. rusticus* for dietary and behavioral analyses in parentheses after the lake‐level CPUE. For the year invaded, all lakes were invaded prior to the initial sampling by Gregory M. Capelli and Magnuson (1983) or earliest initial sampling reported by Larson et al. (2019).

### 2.2 Lake sampling

**FIGURE 2:**
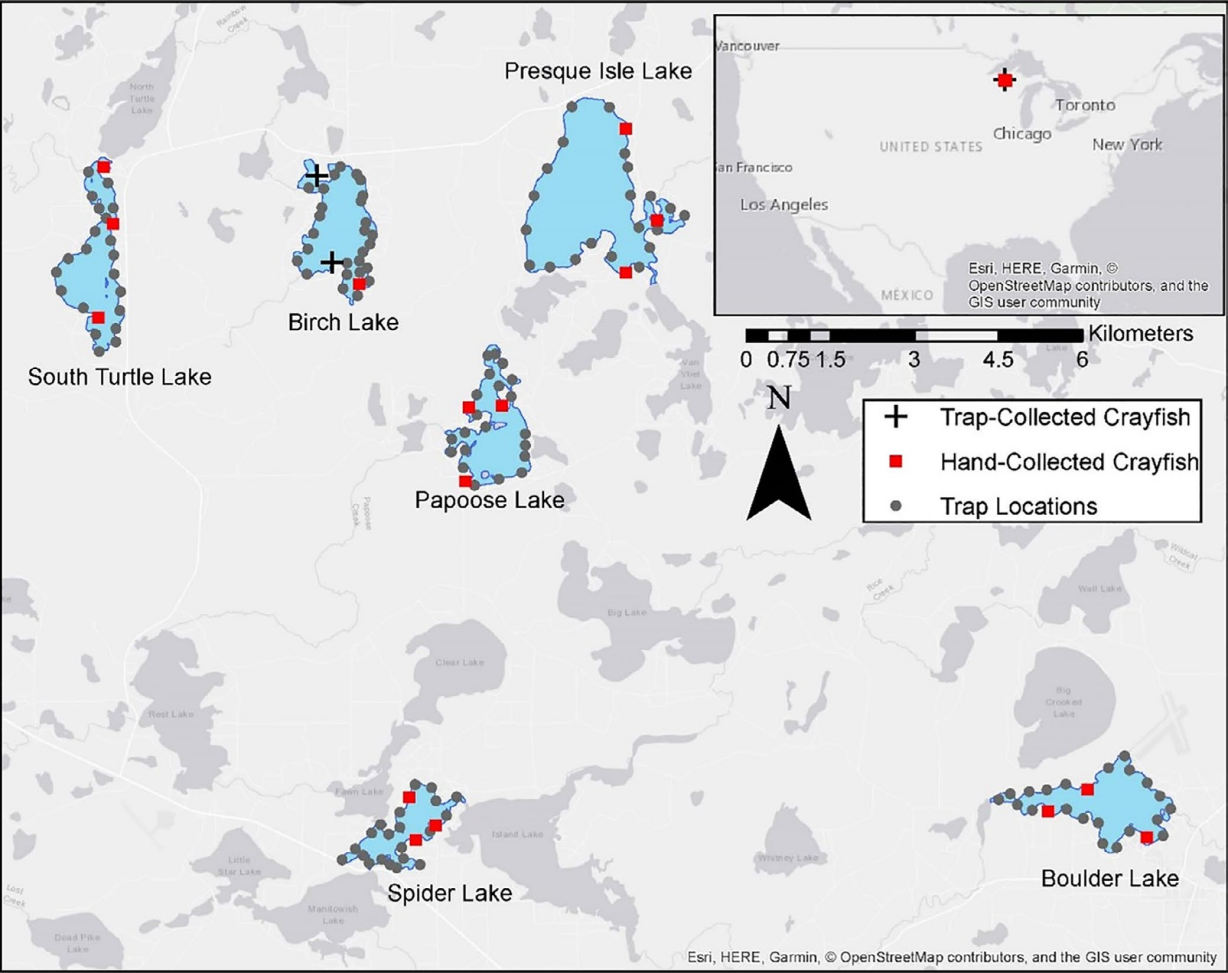
Map of study lakes (blue) in Vilas County, Wisconsin, US. Trap locations for overall estimates of rusty crayfish *Faxonius rusticus* catch‐per‐unit effort (CPUE) are gray circles, and locations of hand‐collection of individuals for behavioral assays and stable isotope analysis are red squares. At Birch Lake, we were unable to hand‐collect *F. rusticus* at two locations due to low abundances; therefore, we trap‐collected *F. rusticus* at these two locations, which are indicated with black plus signs

We sampled our study lakes in July 2018, following convention in this system of estimating crayfish relative abundance as male catch‐per‐unit effort (CPUE) from baited trapping following a mid‐summer molt to reproductively active Form I (Capelli & Magnuson, 1983; Larson et al., 2019). Trap catches in our lakes during this summer period are dominated by male Form I crayfish, although actual sex ratios in these lakes are balanced (1:1 male:female) and male CPUE corresponds well with active search methods for crayfish relative abundance like diver surveys (Capelli & Magnuson, 1983; Olsen et al., 1991). We conducted baited trapping from 17 July 2018 to 23 July 2018 using overnight sets of wire‐mesh, cylindrical “Gee” minnow traps with 0.42 m long by 0.21 m diameter dimensions and 5.0 cm openings on each end of the cylinder. Traps were baited with ~120 g beef liver and set nearshore at depths of 1–3 m. We used 24 traps in all lakes except for Birch Lake, where we used 30 traps following convention of long‐term monitoring in this system (Figure 2; Larson et al., 2019). We then estimated relative abundance as mean male CPUE for each lake, capturing our intended gradient of low to high relative abundance (Table 1).

Following baited trapping, we collected crayfish by hand while snorkeling for use in our behavioral and dietary analyses. Hand‐collection of crayfish avoids biases that may be associated with collecting crayfish by baited trapping, which selects for larger and more aggressive individuals (Larson & Olden, 2016). We collected crayfish by active searching (e.g., overturning substrates like cobble and wood) between 23 July 2018 and 9 August 2018. We collected four crayfish at each of three baited trapping locations (12 crayfish total) dispersed throughout each of our six study lakes in order to represent overall populations of *F*. *rusticus* (Figure 2). In our lowest abundance lake (Birch Lake), we were unable to find crayfish by hand‐collecting at two trap locations. As a consequence, we used baited trapping to collect crayfish from two of the locations at Birch Lake and evaluated behavioral and dietary differences between trapped and hand‐collected crayfish at the one location within Birch Lake where both methods were successful. We found no differences in behavior between trapped and hand‐collected crayfish at this Birch Lake location (Adey, 2019). Across all lakes, we used adult male, Form I *F*. *rusticus* with mean total carapace lengths of 31.1 mm (±3.8 mm *SD*). We used male Form I crayfish because of their higher availability to collection in our lakes during mid‐ to late summer, but future research on this question could also use juvenile and female crayfish.

In addition to crayfish, we collected primary consumers (mussels and snails) to assess potential for differences in the baseline stable isotope ratios (δ^13^C and δ^15^N) of pelagic and littoral benthic energy pathways between these lakes. Specifically, δ^13^C is routinely used to represent dependence of consumers on either pelagic or littoral benthic energy pathways in freshwater lakes, whereas δ^15^N is used to estimate trophic position (Vander Zanden & Rasmussen, 1999). Freshwater mussels have generally depleted (more negative) δ^13^C values that represent the pelagic primary producers they consume (i.e., phytoplankton), freshwater snails have generally enriched (more positive) δ^13^C values that represent the littoral benthic primary producers they consume (i.e., algae or rooted macrophytes), and both mussels and snails provide baseline organisms with a trophic position of two that other consumers can be compared against (Vander Zanden & Rasmussen, 1999). We collected tissue from Unionidae mussels from all six lakes, non‐native Chinese mystery snails *Cipangopaludina chinensis* from four lakes (Birch, Boulder, South Turtle, Spider), and Planorbidae snails from one lake (Presque Isle). We were unable to find any snails in Papoose Lake likely due to consumption by a high abundance *F*. *rusticus* population (McCarthy et al., 2006). Whole primary consumers were kept in water from their respective lakes for transport to the laboratory, where they were frozen until processing for stable isotope analysis.

Following collection, crayfish were transported from their respective lakes to Trout Lake Station (TLS), a field station operated by the Center for Limnology at the University of Wisconsin, Madison, US. Crayfish were kept in separate compartments in tackle boxes during transit to isolate the crayfish from each other. We provided a shallow supply of water in the tackle boxes to keep the crayfish moist during transport to the laboratory. At TLS, the crayfish were kept in their own individual small containers (1 L) to avoid interactions. The containers were filled with water supplied from adjacent Trout Lake, and water was changed every other day. Containers were aerated by air stones connected to air pumps via tubing. We added shelter structures (7.5 cm long × 4.5 cm diameter half PVC pipe) to these containers prior to bringing the crayfish to the laboratory. The crayfish were fed half an algae wafer (~0.3 g; Hikari Tropical Algae Wafers, Himeji, Japan) per day, except when fasting prior to behavioral assays. Past research has demonstrated that *F*. *rusticus* δ^13^C and δ^15^N stable isotope ratios of muscle tissue do not shift significantly toward laboratory foods over similar laboratory feeding durations (Adey & Larson, 2020; Glon et al., 2016b).

### 2.3 Behavioral assays

Six behavioral assays were conducted following a 1‐day acclimation period in the laboratory at TLS. We fasted the crayfish for 24 hr before the diet assays, but no fasting occurred for the remaining assays (Pintor et al., 2008). We conducted assays of shelter use, exploration, feeding flexibility (with three separate assays), and response to stimuli to broadly characterize behavioral individual specialization of *F*. *rusticus* individuals. These behavioral assays were chosen to represent four of the five temperament traits in animal behavior: boldness, exploration, activity, and aggressiveness (Réale et al., 2007).

The first behavioral assay was conducted in the 1‐L containers where crayfish were maintained during their time in the laboratory, while the five remaining assays (exploration, three feeding trials, and response to stimuli) were all conducted in separate 5.5‐L experimental arenas (36 cm length × 23 cm width × 10 cm height). For each of these five assays in experimental arenas, the water was changed between each crayfish observation, and each individual was acclimated for 15 min prior to the start of the assay. We used black plastic sheeting to create a blind to obscure the observer from the crayfish.

The first behavioral assay measured the activity and exploration of individuals. For this assay, we observed shelter occupancy by crayfish over 12 hr following the 1‐day acclimation period, as a measure of activity. Less active crayfish were anticipated to remain in shelters throughout the day, whereas more active crayfish were anticipated to explore. During the 12‐hr period from 8 a.m. to 8 p.m. an observer recorded whether the crayfish was within the shelter hourly.

The second behavioral assay measured exploration by assessing the willingness of crayfish to explore a new area (Verbeek et al., 1994). We placed an opaque, black plastic divider in the experimental arena to block a portion of the tank from view of the crayfish during the 15‐min acclimation period. After this period, the opaque divider was removed, and the observer recorded the latency time it took for the crayfish to explore the new area with a maximum time of 20 min.

The third, fourth, and fifth behavioral assays consisted of three separate feeding trials related to individual flexibility in feeding and boldness. For each of these assays, the experimental arena was divided into three sections, separated by two plexiglass dividers. The first and second feeding assays looked at feeding flexibility, in which the first assessed the time it took for crayfish to take a high‐quality food item (snail), while the second assessed the time it took for the crayfish to take a lower quality food item (detritus). For each of these feeding assays, the first section of the experimental arena initially contained the individual being observed, the second section of the container contained the food item, and the third section of the container was empty. We used *C. chinensis* (15.9 ± 3.02 mm; mean ± *SD*) from Allequash Lake in Vilas County, Wisconsin, US, as our high‐quality food item (“snail”) and conditioned Red Oak *Quercus rubra* leaves collected from Presque Isle Lake as our low‐quality food items (“detritus”; Nystrom et al., 1999). Leaves were cut into 10 mm squares. After the 15‐min acclimation period, the divider between the crayfish and the food item was removed. The observer recorded the time until the crayfish first attempted to feed on the item.

The third feeding assay measured the boldness of the individual to feed in the presence of a conspecific individual. We replicated the snail feeding assay above, but with a size‐matched conspecific *F*. *rusticus* present in the third (previously empty) section of the arena. Size‐matched conspecifics were within a mean of 3.9 mm (± 3.87 mm *SD*) total carapace length of the study individuals. The clear dividers permitted the crayfish to see each other, but not to physically interact, while the study individual foraged. As in the food quality feeding assays, the divider between the study crayfish and the snail was removed after the 15‐min acclimation period, and the observer recorded the time until the crayfish first attempted to feed on the item. In each of the three feeding assays, we recorded the latency to feed with a maximum time of 20 min (1,200 s).

The sixth behavioral assay examined the aggressiveness of individuals. To test the fight or flight responses of individuals, we observed their response to a novel object moving toward them (Pagé & Cooper, 2004). We chose to use a novel object instead of recording interactions with a conspecific to limit the effect or dependency of the conspecific's behavior on the outcome of this assay. After the crayfish acclimated for 15 min in the experimental arena unconstrained by any dividers, the observer moved a wooden rod (29 cm length × 0.25 cm diameter) toward the crayfish. For consistency, we approached the anterior side of the crayfish with the wooden rod in each trial and scored their initial response. We used a truncated version of the scale from Bruski and Dunham (1987), which ranges from −2 to 4 with negative scores indicating a degree of retreat from the rod, positive scores indicate an aggressive response such as threat displays and grabbing, and zero indicating no response (Table 2). This assay was conducted three times for each crayfish with a 15‐min re‐acclimation period between each approach with the rod. We then summed the scores for each crayfish across the three approaches for subsequent statistical analyses. We summed rather than averaged aggressiveness scores because these two approaches were monotonic in comparison, but summed scores better reflected repeatability of behaviors by our crayfish (i.e., as a broader range of values between low vs. high aggressiveness).

**TABLE 2:** Responses of *Faxonius rusticus* to approach by a novel object with associated scores for calculating degree of aggression (Bruski & Dunham, 1987)

Score	Action
−2	Tail flip or fast retreat
−1	Slow retreat
0	Within one body length with no visible interaction
1	Approach without threat display
2	Approach with threat display (e.g., meral spread, antennal whips)
3	Boxing, pushing, or other agonistic interaction with closed chelae
4	Grabbing, tearing, or other agonistic interaction with open chelae

### 2.4 Stable isotope processing

We acclimated crayfish for 1 day in the laboratory prior to starting behavioral assays, as opposed to a more customary 1‐week acclimation period (e.g., Daws et al., 2002; Schneider et al., 2001), in order to limit the amount of crayfish isotopic shift away from their field diet and toward the laboratory diet. Recent work has shown acclimation time differences between 1 day and 1 week minimally affect comparisons of *F*. *rusticus* behavior and diet within an individual population (Adey & Larson, 2020). Further, we used muscle tissue for our stable isotope analysis because crayfish muscle tissue has slow turnover rates relative to other tissues like hepatopancreas or hemolymph (Adey & Larson, 2020). *Faxonius rusticus* muscle tissue has half‐lives for δ^13^C and δ^15^N of approximately 30 days, reaching equilibrium with diet after four or five half‐lives (Glon et al., 2016b). Accordingly, our relatively short duration of laboratory feeding would not shift *F*. *rusticus* δ^13^C and δ^15^N values away from their field diets. Crayfish were frozen immediately following the aggression assay to preserve their tissue until preparation for stable isotope analysis.

Muscle tissue from the crayfish, and foot tissue of both primary consumers (snails and mussels), was dissected for stable isotope analysis, after which all samples were placed in a drying oven (Fisher Scientific Isotemp 100 L Oven) at 60℃ for 24 hr. Dried samples were homogenized using a stainless‐steel mortar and pestle that was cleaned between each sample. We then used a microbalance to weigh homogenized tissue into tins (UC‐Davis Stable Isotope Facility recommendation for animal tissues: 1 ± 0.2 mg). We next shipped samples to the University of California‐Davis Stable Isotope Facility, where they were analyzed using a PDZ Europa ANCA‐GSL elemental analyzer with a PDZ Europa 20‐20 isotope ratio mass spectrometer (Sercon Ltd., Cheshire, UK). The sample values were corrected using laboratory reference standards, which have a long‐term standard deviation of 0.2‰ for carbon and 0.3‰ for nitrogen.

### 2.5 Statistical analysis

Stable isotopes can be used to infer individual diet specialization by a variety of measures that calculate the breadth of population niches (Jackson et al., 2011; Layman et al., 2007). More similar diets of individuals within a population will have similar or clustered δ^13^C and δ^15^N values, whereas less similar diets of individuals within a population will have more dispersed or variable δ^13^C and δ^15^N values. As stable isotopes integrate diet continuously over time for individuals (a repeated measure), large population niche widths in this case correspond to higher individual dietary specialization, whereas smaller population niche widths correspond to lower individual dietary specialization (Araújo et al., 2007; Bolnick et al., 2003). To quantify individual specialization by diet for *F*. *rusticus* within our study lake lakes, we used values of δ^15^N and δ^13^C to calculate 95% confidence ellipses using the ggplot2 package (version 3.3.2; Wickham, 2016) in R version (3.5.2). Although most stable isotope studies use the SIBER package (Jackson et al., 2011) for this purpose, we used 95% confidence ellipses for ggplot2 for consistency in the measure of individual specialization between our diet and behavior metrics (below). We performed our analyses with uncorrected δ^13^C and δ^15^N, rather than standardizing to trophic position or δ^13^C width of primary consumers per lake as in papers like Araújo et al. (2007) or Larson et al. (2017), because our results were insensitive to the choice to use uncorrected versus standardized stable isotope ratios in our analyses. For example, the 95% confidence ellipses of uncorrected stable isotope ratios for *F*. *rusticus* were highly correlated (*R* = 0.79) with this same measure of individual specialization when standardizing 95% confidence ellipses to the δ^13^C values of primary consumers in each lake.

To assess the amount of individual specialization by behavior within each lake, we conducted a scaled principal component analysis (PCA) using the correlation matrix through the vegan package (version 2.5‐6; Oksanen et al., 2019) in R on all of our behavioral responses. Based on the variance explained in our first two axes and the marginal significance of the third axis, we quantified individual specialization in behavior within populations by calculating 95% confidence ellipses on the first two axes using the ggplot2 package (Wickham, 2016) in R.

We used linear models to fit linear and quadratic relationships between relative abundance (CPUE from baited trapping) and individual specialization as the area of 95% confidence ellipses from both diet and behavior measures using Base R. Linear fits could represent increasing or decreasing individual specialization in response to relative abundance. Quadratic fits were anticipated to indicate a positive unimodal relationship between individual specialization and relative abundance, although these fits could also reflect a negative unimodal relationship where individual specialization was lower at intermediate relative abundances. We then used likelihood‐ratio tests to compare the linear and quadratic models for both dietary and behavioral specialization to each other, as well as to a null model (intercept only), with the lmtest package (version 0.9‐37; Zeileis & Hothorn, 2002) in R. We interpret the more parsimonious (i.e., simpler) of these nested models as preferable for interpretation in cases where likelihood‐ratio tests fail to find significant differences between models.

## 3 RESULTS

**FIGURE 3:**
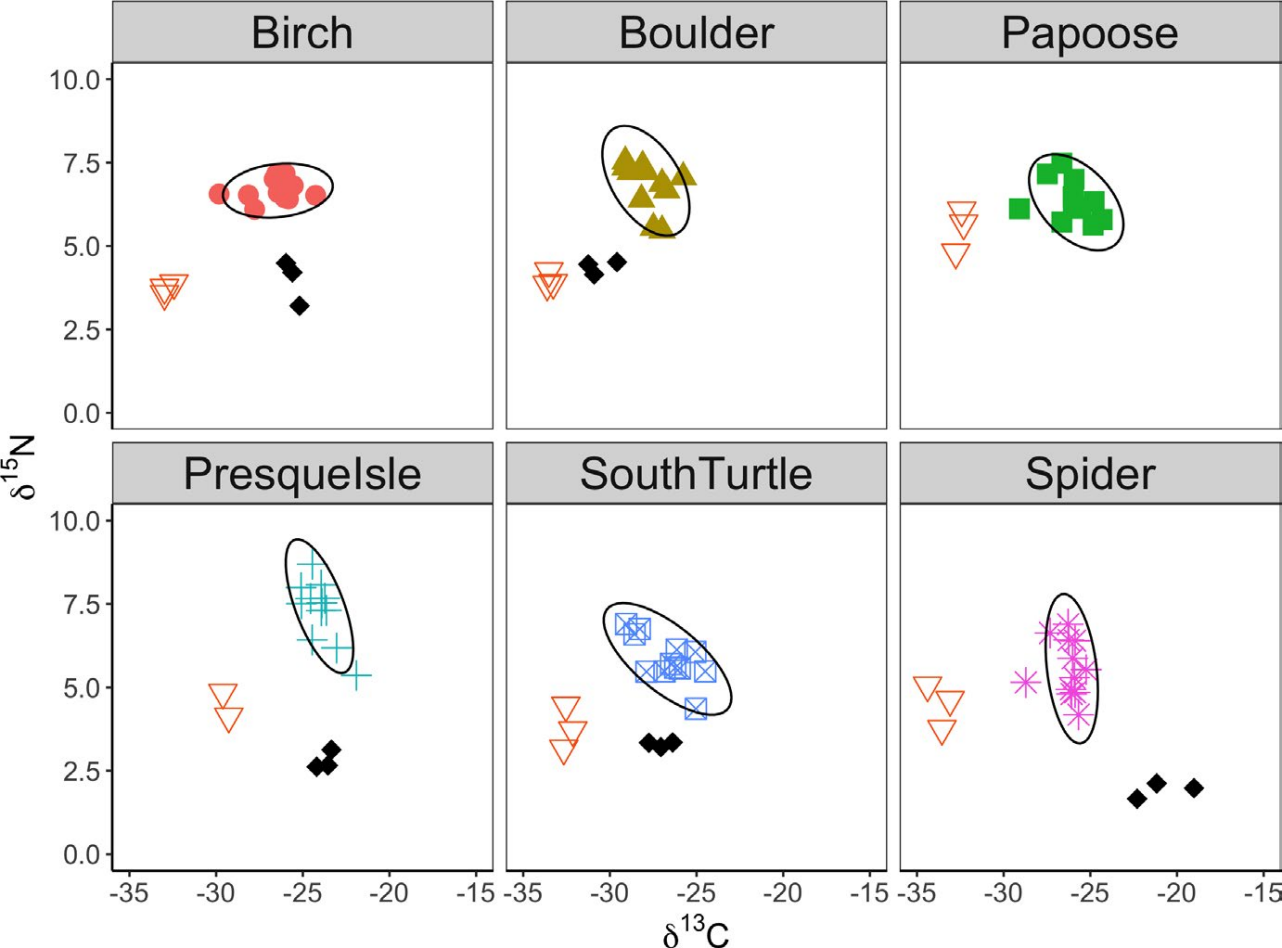
Stable isotope data for mussels (inverted triangles), snails (black diamonds), and rusty crayfish *Faxonius rusticus* (with 95% confidence ellipses) for each lake (unique symbols per lake). We collected Unionidae mussels from all lakes; Chinese mystery snails *Cipangopaludina chinensis* from Birch Lake, Boulder Lake, South Turtle Lake, and Spider Lake; and Planorbidae snails from Presque Isle Lake. We were unable to find any snails at Papoose Lake

Benthic littoral and pelagic energy sources appeared largely consistent between our study lakes, with Unionidae mussels depleted in δ^13^C, reflecting pelagic food webs, and snails enriched in δ^13^C, reflecting benthic littoral food webs (Figure 3). Crayfish were mostly intermediate in δ^13^C values relative to primary consumers and were consistently more enriched in δ^15^N, indicating that they were higher in trophic position than either mussels or snails. Stable isotope ellipses of crayfish populations were variable between lakes, suggesting differences in the magnitude of individual dietary specialization. Some populations (e.g., Birch) exhibited relatively small 95% confidence ellipses, indicating less individual specialized by diet, and some populations exhibited relatively large 95% confidence ellipses (e.g., South Turtle), indicating more individual specialization by diet (Figure 3).

**FIGURE 4:**
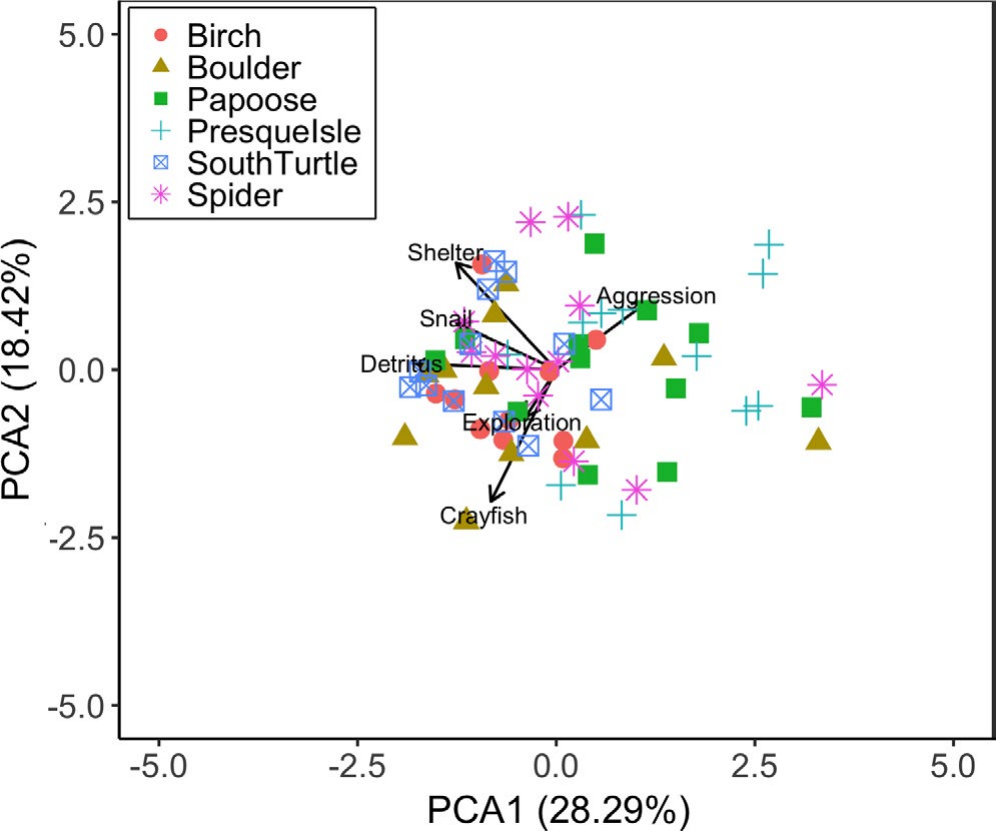
Principal component analysis (PCA) on rusty crayfish *Faxonius rusticus* laboratory behaviors, with vectors corresponding to the six behavioral assays: shelter occupancy (Shelter), latency to explore (Exploration), latency to feed on a snail (Snail), latency to feed on conditioned leaf litter (Detritus), latency to feed on a snail in the presence of another crayfish (Crayfish), and aggressiveness of response to stimuli (Aggression). Higher values for Exploration, Snail, Detritus, and Crayfish are associated with individuals that were slow to, or did not, explore and feed. Individual crayfish are labeled by lake consistent with other figures

**FIGURE 5:**
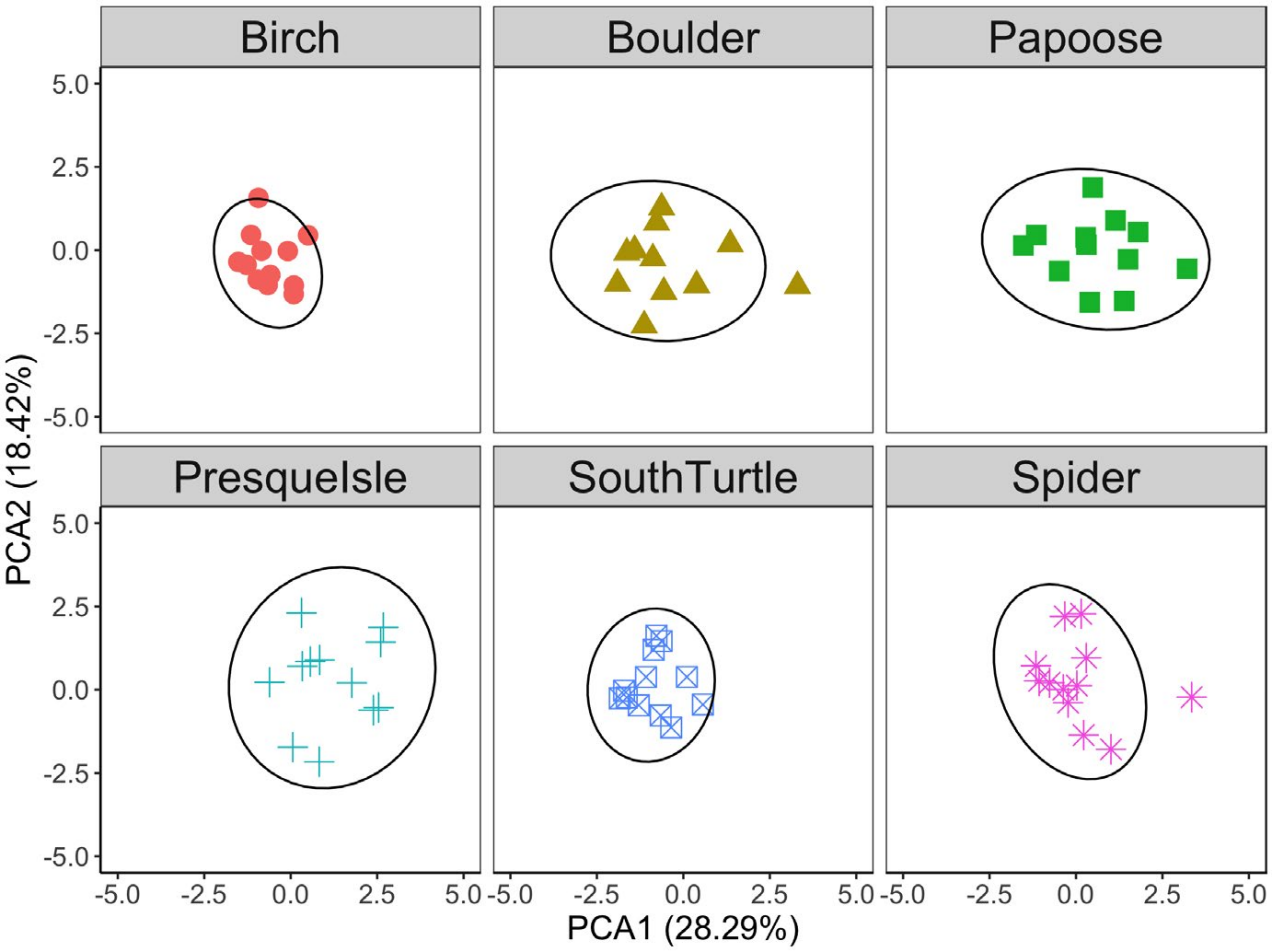
Principal component analysis (PCA; Figure 4) with 95% confidence ellipses for the rusty crayfish *Faxonius rusticus* behavioral assays with each of the six lakes as its own panel

The first two axes of our PCA explained 46.71% of the variance in crayfish behavior and were significant by Kaiser's rule (*SD* = 1.30, 1.05), whereas the third axis was marginally significant (*SD* = 1.01). The first axis explained 28.29% of the variance, and the second axis explained 18.42% of the variance (Figures 4 and 5). For the first axis of the PCA, positive loadings were associated with crayfish that were more aggressive toward the novel object, while negative loadings were associated with crayfish that spent more time in shelter, were slow to feed in all three feeding assays, and did not explore the novel area. As such, the first axis of our PCA may represent a behavioral syndrome of correlated behaviors, in which shy individuals are represented by negative values and bold individuals are represented by positive values on this PCA. For the second axis of the PCA, crayfish with positive loadings spent more time in shelter, were slow to feed on the snail and detritus, and were more aggressive, while negative loadings were associated with crayfish that did not explore and that did not feed in the presence of another crayfish.

**FIGURE 6:**
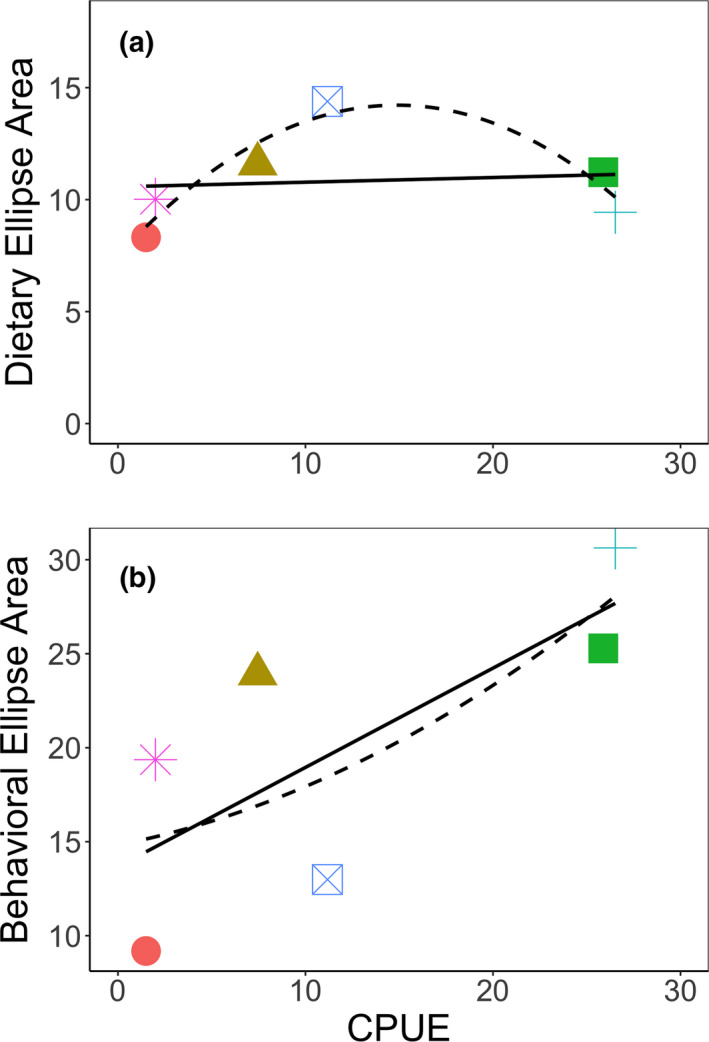
Linear and quadratic models for individual dietary specialization (a) and individual behavioral specialization (b) of rusty crayfish *Faxonius rusticus* relative to catch‐per‐unit effort (CPUE; Table 1). Measures of individual specialization within lakes are 95% confidence ellipses from stable isotope analysis of diet (Figure 3) or principal component analysis on laboratory behavior (Figure 5). Symbols correspond with study lakes in Figures 3, 4, 5

We found a significant difference between the quadratic and linear models for the relationship between individual specialization of diet and relative abundance (χ^2^ = 13.91, *df* = 1, *p* < .001), as well as a significant difference between the quadratic and null models (χ^2^ = 11.81, *df* = 2, *p* = .003). We found no significant difference between the linear and null model for this comparison (χ^2^ = 0.08, *df* = 1, *p* = .784). Accordingly, we interpret the quadratic model as more supported, and this model fits the data relatively well with an adjusted *R* ^2^ of 0.77 (Figure 6). We found no significant difference between the quadratic and linear models for the relationship between individual specialization of behavior and relative abundance (χ^2^ = 0.11, *df* = 1, *p* = .739) and no significant difference between the quadratic and null models (χ^2^ = 4.89, *df* = 2, *p* = .087). Further, the quadratic model in this case fits a weakly negative relationship between behavioral specialization and intermediate relative abundances, contrary to our a priori prediction. However, we did find a significant difference between the linear and null models of behavioral specialization and relative abundance (χ^2^ = 4.75, *df* = 1, *p* = .029). We interpret the linear model for this relationship as more supported, and this model had an adjusted *R* ^2^ of 0.44 (Figure 6).

## 4 DISCUSSION

Our study was motivated by Toscano et al.'s (2016) call to unify and explore linkages between intraspecific variation in behavior and diet, as well as Glon et al.'s (2016a) identification of a methodological approach for this purpose. Consistent with our expectations, we found a unimodal relationship between relative abundance and individual specialization of diet, where specialization was highest at intermediate relative abundances but declined at high relative abundances. This suggests our study organism *F*. *rusticus* does experience density‐dependent effects of limited food resources at high relative abundances in these lakes (Lodge et al., 1994; Wilson et al., 2004). Alternatively, we found a weak but positive linear relationship between relative abundance and individual specialization of behavior for this crayfish. Because we used the same individuals to measure both dietary and behavioral individual specialization concurrently, the difference in relationships found can be attributed to the measure of individual specialization rather than differences between individuals or populations (Adey & Larson, 2020; Glon et al., 2016a).

A unimodal relationship between intraspecific competition and individual specialization of diet is dependent on resource depletion at high densities, which is not seen for all species under in situ conditions. For example, Svanbäck and Persson (2004) found that European perch *Perca fluviatilis* dietary specialization increased with increasing density of this fish, with no decrease in specialization at higher abundances. Alternatively, we observed decreased dietary specialization at our highest *F*. *rusticus* relative abundances. At low abundances, *F*. *rusticus* likely feeds preferentially on high‐quality animal prey (i.e., snails) and therefore has low individual dietary specialization. At intermediate abundances, some *F*. *rusticus* individuals may specialize to exploit a broader selection of diet items, like primary producers or lower quality detritus, as high‐quality food becomes scarce. At very high abundances, *F*. *rusticus* may altogether deplete high‐quality resources, resulting in a contraction of individual specialization to lower quality or less preferred food. Our unimodal relationship between *F*. *rusticus* dietary specialization and relative abundance is highly consistent with past work on this invasive crayfish, which has found hyperabundant populations of *F*. *rusticus* to strongly reduce the abundance and richness of benthic invertebrates such as snails in our study lakes (e.g., McCarthy et al., 2006; Roth et al., 2006; Wilson et al., 2004). As such, our results support past studies that have found negative relationships between organismal density and individual diet specialization in cases where species can deplete some food resources (Jones & Post, 2013; Schindler et al., 1997). Future studies of dietary specialization in *F*. *rusticus* could also measure and relate prey availability or diversity directly to individual diet specialization of this crayfish to further link our observed pattern to mechanism (Bolnick & Ballare, 2020).

We found a positive linear relationship between individual specialization of behavior and relative abundance for *F*. *rusticus* across an abundance gradient. In particular, we observed more bold and aggressive individuals in our high than low abundance lakes, similar to the increase in these behaviors observed with increased population densities of another crayfish species, *P. leniusculus* (Pintor et al., 2009). In high‐density populations, behaviors such as aggression and boldness can be commonly correlated in behavioral syndromes necessary for successful intraspecific competition (Dingemanse et al., 2007; Sih et al., 2004). However, not all individuals in our high abundance lakes exhibited increased aggression or boldness, allowing for an increase in individual specialization of behavior within populations (an increase in ellipse area) rather than just a change in the most common behaviors exhibited (a shift in ellipse space), or even a decrease in individual specialization of behavior as a behavioral syndrome perhaps dominates at high relative abundances. The incidence of highly aggressive and bold individuals might be moderated in our most high abundance *F*. *rusticus* lakes relative to some other crayfish study systems (e.g., Pintor et al., 2009) if these behaviors not only confer benefits for intraspecific competition but also carry risks related to increased predation by fish or other consumers (DiDonato & Lodge, 1993; Roth & Kitchell, 2005). Finally, the linear relationship we found between relative abundance and individual specialization of behavior for *F*. *rusticus* was weaker than the unimodal relationship we found for individual specialization of diet and would benefit from greater statistical power or replication of additional populations in future studies.

We have attributed differences in individual specialization of *F*. *rusticus* within our study lakes to the effects of increasing intraspecific competition at high relative abundances, but other factors such as top‐down effects of predators or bottom‐up effects of resource availability could be affecting both crayfish behavior and diets (Evangelista et al., 2019; Raffard et al., 2020). We chose our lakes to be as similar as possible in terms of size, productivity, and *F*. *rusticus* invasion history while still maintaining our gradient of relative abundance, but these lakes inevitably have some abiotic and biotic differences. First, although fish may alter the diet and behavior of crayfish (Keller & Moore, 2000; Stein & Magnuson, 1976), our lakes have broadly similar fish communities (Kreps et al., 2016). However, we do not know the relative abundances of these fish species concurrent with our sampling for *F*. *rusticus* to infer how they might influence crayfish behavior and diet. For example, crayfish in temperate lakes may be less active when predatory fish are highly abundant (Jurcak et al., 2016). Further, some of our lakes do differ by water clarity, which has been shown to impact foraging behavior and diet choice for some freshwater taxa (e.g., Crowl, 1989; Wong & Candolin, 2015). Crayfish respond to chemical and visual cues both behaviorally and in feeding decisions (Correia & Anastácio, 2008; Renai & Gherardi, 2004). Future studies investigating individual specialization of crayfish diet and behavior might benefit from having more similar habitat characteristics between populations, such as clarity or productivity, or could control for these habitat covariates in models with increased replication of populations. Finally, recent research from our study region has suggested that behavior of these crayfish can be strongly influenced by trematode parasites, where *F*. *rusticus* individuals without these parasites are more likely to forage when predators are present and have increased growth rates (Sargent et al., 2014). We estimated trematode parasite loads in the hepatopancreas of all *F*. *rusticus* individuals used in our study and found similar infection rates of our crayfish regardless of lake (Adey, 2019). Accordingly, we do not expect that results of our study were strongly influenced by behaviors moderated by trematode parasites.

Our study used stable isotope analysis on individual crayfish assayed for behavior in the laboratory to test whether the relationship between individual specialization and intraspecific competition is contingent on the metric of individual specialization used. We found a unimodal relationship between individual specialization of diet and intraspecific competition, in which resource depletion at high relative abundances likely restricts or limits the potential for continuing individual specialization. Alternatively, we found that individual specialization of behavior, which is not necessarily resource‐limited, increases linearly with increasing relative abundance. Future studies on this topic could apply our methodology, or related approaches (i.e., fatty acid analysis), to other systems and similar questions (Galloway et al., 2014; Iverson et al., 2004). In particular, our results would benefit from greater replication and associated statistical power, which might be possible in systems where collection of individuals and behavioral observations are easier across many sites or populations (i.e., smaller arthropods; Gatti et al., 2020). The relationship between intraspecific competition and individual specialization has an important role in both ecology and evolution, and we demonstrate here that stable isotopes and laboratory assays have the potential to unite the study of individual variation across both diet and behavior (Glon et al., 2016a; Toscano et al., 2016).

## CONFLICT OF INTEREST

None declared.

## AUTHOR CONTRIBUTIONS

**Amaryllis K. Adey:** Conceptualization (lead); data curation (equal); formal analysis (lead); methodology (equal); validation (equal); visualization (lead); writing‐original draft (lead); writing‐review & editing (equal). **Eric R. Larson:** Conceptualization (supporting); data curation (equal); formal analysis (supporting); funding acquisition (lead); methodology (equal); project administration (lead); resources (lead); supervision (lead); validation (equal); visualization (equal); writing‐review & editing (equal).

## Data Availability

Data are available at the University of Illinois Data Bank (https://doi.org/10.13012/B2IDB‐8355786_V1).
